# Perceived barriers and facilitators to uptake of non-traditional roles by pharmacists in Saudi Arabia and implications for COVID-19 pandemic and beyond: a qualitative study using Theoretical Domain Framework

**DOI:** 10.1186/s40545-021-00307-w

**Published:** 2021-02-24

**Authors:** Eeman Mohammed, Saval Khanal, Zahraa Jalal, Ejaz Cheema, Mohammed H. Abutaleb, Vibhu Paudyal

**Affiliations:** 1 School of Pharmacy, Institute of Clinical Sciences, College of Medical and Dental Sciences, Sir Robert Aitken Institute for Medical Research, University of Birmingham, Birmingham, B15 2TT UK; 2 Behavioral Science Group, Warwick Business School, University of Warwick, Coventry, UK; 3 Pharmacy Department, King Fahad Central Hospital-Jazan Health Affairs, Ministry of Health, Jazan, Saudi Arabia

**Keywords:** Non-traditional roles, Saudi Arabia, Clinical, Pharmacist, Theoretical domains framework (TDF)

## Abstract

**Background:**

The COVID-19 pandemic has further strengthened the need for pharmacists to uptake non-traditional roles. Pharmacy practice in Saudi Arabia is emerging in recent years with greater policy emphasis on pharmacists taking new clinical roles. This study aimed to explore the experiences, perceptions and barriers of Saudi pharmacists about their uptake of non-traditional roles using Theoretical Domains Framework (TDF).

**Methods:**

A qualitative semi-structured study using face-to-face or telephone interviews were conducted. Eligible participants included qualified pharmacists from Saudi Arabia. Interviews focused on pharmacist’s perceptions, current opportunities and key challenges towards the uptake of non-traditional roles. Interviews were audiotaped and transcribed verbatim. Results were analysed through the framework analysis method and were later mapped with respective domains of TDF.

**Results:**

A total of 14 pharmacists completed the interview (9 females and 5 males). Participants showed an overall positive attitude towards the uptake of non-traditional roles. Participants felt that there was wider support available for pharmacists at the policy level to uptake non-traditional roles. However, a need for greater recognition of roles by other healthcare professionals and patients were identified. Participants alluded to reluctance of some physicians to take on board the suggestions from a pharmacist. Key barriers to uptake of non-traditional roles were related to environmental context and resources domain of TDF. For example, participants discussed the need for even further practical experiences during their undergraduate degree to become ready to adopt non-traditional roles in clinical practice.

**Conclusions:**

Participants of this theoretically informed qualitative study showed an overall positive attitude towards the way pharmacy practice is progressing in Saudi Arabia and their uptake of non-traditional roles. However, there is a need to improve interdisciplinary working, patient awareness of pharmacist competencies and their educational preparedness in furthering their uptake of non-traditional roles. Addressing such barriers and promoting uptake of novel roles by pharmacists is imperative in the context of emerging COVID-19 and future pandemics.

## Introduction

The COVID-19 pandemic has further strengthened the need for pharmacists to uptake non-traditional roles. The healthcare system in Saudi Arabia has aimed to evolve significantly over the last few decades brought by considerable changes in the quality of health and social care services including pharmacy services. With the growing population and increase in life expectancy, the Ministry of Health (MOH) continues to look for new ways to develop the healthcare system that is well suited to meet the current and future needs of Saudi population. In the late 2016, the MOH provided over 40 health care initiatives as part of the Saudi Vision 2030. Of these 40 initiatives, 15 were related to the advancement of pharmacy practice and pharmaceutical care [1].

Currently, community pharmacy in Saudi Arabia has the largest workforce of pharmacists [2]. Pharmacists working in the community sector are primarily responsible for dispensing and provision of counselling to patients on both prescription and over-the-counter (OTC) medicines [2]. Hospital pharmacy on the other hand offers diverse opportunities for pharmacists in Saudi Arabia that extends beyond dispensing and preparing medications to ensuring safe and effective use of medicines through provision of patient counselling and drug therapy monitoring [3]. Pharmacy graduates may also choose to work in other health care sectors in either public services run by the government or in private sectors such as the university affiliated hospitals, medical services in security or military affairs, and private medical institutions. Other regulatory organisation such as the Saudi Drug and Food Authority, the Saudi Center for Disease Prevention and Control can also be work place for Saudi pharmacists [4].

Pharmacy practice in Saudi Arabia is advancing with the role of pharmacists becoming more patient-centred and clinically focused as shown with the introduction of PharmD programme in Saudi Arabia in 2005 [4]. The curriculum of the PharmD programme in Saudi universities is mainly based on American PharmD curriculum through a collaborative agreement with an Accreditation Council for Pharmacy Education (ACPE) accredited programme, with limited modifications aimed to reflect the culture, and pharmacy administration and law. The aim of this movement was to produce competent pharmaceutical care providers to provide an integrated course to help students develop necessary skills to practise patient-orientated and clinical roles that is in line with the Saudi 2030 vision initiatives set out by the MOH [5]. Such programme utilised different learning methods such as team- based learning (TBL), problem-based learning (PBL), or active learning to help students develop their clinical skills and knowledge in order to be easily accommodated by the healthcare system [6].

The introduction of new curricula in pharmacy schools has shifted the focus of pharmacists from the traditional roles of medicines’ compounding and distribution towards patient-centred clinical roles. However, the extent to which these roles have been implemented in Saudi Arabia is unknown. Previous research conducted in Saudi Arabia have demonstrated that pharmacists have the potential to improve patient health outcomes directly in hospital and community settings and indirectly through pharmaceutical industry and practice-based research [7–20]. However, to authors’ knowledge, no study to date has been conducted that has explored the pharmacists’ perception about the extended roles in pharmacy in Saudi Arabia. In addition there is a lack of theory based research in identifying key barriers and facilitators to uptake of non-traditional roles. This study therefore aims to explore the perceptions of Saudi pharmacists about the uptake of non-traditional roles in pharmacy using Theoretical Domains Framework (TDF). The TDF includes 14 domains covering 84 theoretical constructs [21]. The 14 domains include knowledge, skills, resources, social influences and intentions. TDF enables the identification of appropriate components of planned behavioural interventions, the barriers and enablers which need to be addressed, and the way behaviour changes brought through the interventions can be measured and understood [22–26]. TDF has been recently extensively used in pharmacy practice research [27–29] including their perceptions about extended clinical pharmacy services [30].

## Method

This is a qualitative study conducted with the Saudi pharmacists to identify the perceived barriers and enablers to update the non-traditional role in their country. Eligible participants were recruited using convenience sample of qualified Saudi pharmacists who were in the UK as part of their further study. This cohort was purposively selected as these group of pharmacists were considered to know non-traditional roles of pharmacists in other countries (i.e. UK) and the current status of pharmacy practice in their country. Participants were recruited through circulation of invitations to participate in postgraduate forum of a pharmacy higher education institution and snowball sampling technique. An in-depth semi-structured face-to-face or telephone interviews lasting approximately 30 min were conducted. An interview guide comprising of 18 questions was developed by the members of the research team (Table 1). The interview guide was piloted among four final year MPharm students at the University of Birmingham to determine the clarity, ease of understanding of questions and interview duration. Participants who gave their consent to take part in the study were interviewed and audio recorded. The audio recordings were transcribed verbatim. The interview was conducted by in English language.

**Table 1:** Interview schedule

S.N.	Questions	Any prompts/ follow up questions
1	Can I get you to just briefly describe your area of expertise and how long you have been qualified for?	
2	What areas can pharmacists in Saudi Arabia specialise into/what routes can they take?	
3	What do you think patients expect from pharmacists in Saudi Arabia?	
4	If I asked you to define the current role of a pharmacist, what would you say?	Prompt: How does this compares with the roles about a decade ago)
5	Can you give me your definition of ‘traditional’ and non-traditional roles in pharmacy practice?	
6	Can you give me an example of a service that you would consider non-traditional?	Prompt: what other services do pharmacists provide other than dispensing prescriptions (i.e. vaccinations, OTC counselling, private counselling, blood pressure monitoring etc.)
7	How do you feel about how pharmacy practice is changing in Saudi Arabia?	
8	Do you that think that the roles of pharmacists is becoming more clinical?	Follow up: How do you feel this has benefited patients?
9	Do you think pharmacy education in Saudi Arabia is fit for purpose to enable new graduates to uptake non-traditional roles?	
10	Do pharmacists have adequate continuous professional development opportunities to uptake non-traditional roles?	
11	How do pharmacists feel about the wider support to uptake non-traditional roles?	Prompt: support from government, employers, patient perceptions etc
12	Do you feel patients value pharmacists uptaking non-traditional roles?	
13	What are your perceptions of a pharmacists’ role in the healthcare team (a multi-disciplinary team of health and social care professionals who are the first point of contact for patients)?	
14	How do you think pharmacists work with other healthcare professionals?	
15	How is this changing with the uptake of non-traditional roles?	
16	Can you tell me about a new and innovative service that you have provided in recently in your field?	
17	Can you tell me what you think are the key barriers to the uptake of non-traditional roles?	
18	What do you think are the key challenges towards the provision of non-traditional services pharmacy roles in Saudi Arabia?	
19	Do you have any comments to add?	

Data analysis for the transcribed data was done through the framework analysis method by using the inductive approach to identify new themes and by deductive approach to identify themes that may have been pre-conceptualised based on previous literature [31]. Subsequently, identified themes were triangulated with the domains in Theoretical Domain Framework (Table 2) [21]. Two researchers independently assessed the domains involved with the themes, and discrepancies were solved by mutual agreement.

**Table 2:** Theoretical Domain Framework

TDF Domain	Definition
Knowledge	An awareness of existence of something
Skills	An ability or proficiency acquired through practice
Social/professional role and identity	A coherent set of behaviors and displayed personal qualities of an individual in a social or work setting
Belief about capabilities	Acceptance of the truth, reality, or validity about an ability, talent, or facility that a person can put into constructive use
Optimism	The confidence that things will happen for the best or that desired goals will be obtained
Belief about consequences	Acceptance of truth, reality or validity about outcomes of behavior in a given situation
Reinforcement	Increasing the probability of a response by arranging a dependent relationship, or contingency, between the response and given stimuli
Intentions	A conscious decision to perform a behavior or a resolve to act in a certain way
Goals	Mental representations of outcomes or end states that an individual wants to achieve
Memory, attention and decision processes	The ability to retain information, focus selectively on aspects of environment, and choose between two or more alternatives
Environmental context and resources	Any circumstance of a person’s situation or environment that discourages or encourages the development of skills and abilities, independence, social competence, and adaptive behavior
Social influences	Those interpersonal processes that can cause individuals to change their thoughts, feelings, or behavior
Emotions	A complex reaction pattern, involving experiential, behavioral and physiological elements by which the individual attempts to deal with a personally significant matter or event
Behavioral regulation	Anything aimed at managing or changing objectively observed or measured actions

Adapted from [19]

## Results

### Demographic characteristics

Fourteen participants (9 female and 5 male) were included in the study (age range 23–39 years). The included participants practiced in a diverse range of settings with majority working as hospital pharmacists, further information of about demography of the participants is in Table 3.

**Table 3:** Demographic profile of the participants

SN	Gender	Current practice setting and/or job title	Length of pharmacy practice
1	Female	Academia	3–4 years
2	Male	Academia	5 years
3	Male	Academia	7 years
4	Female	Academia	8 years
5	Female	Academia	6 months
6	Female	Hospital pharmacist	6 years
7	Female	Hospital pharmacist	2 years
8	Male	Primary care	8 years
9	Female	Military hospital pharmacist	7 years
10	Female	Hospital pharmacist	3 years
11	Female	IV pharmacist	7 years
12	Female	Clinical trials pharmacist, Saudi FDA	2 years
13	Male	Senior pharmacist, Saudi FDA	10 years
14	Male	Senior hospital pharmacist, Security forces hospital	13 years

### Key themes and summary of the findings

Altogether, we identified 16 key themes and subdivided them into 26 subthemes. The identified themes are presented in Table 4. The Table 4 provides a summary of the key themes and sub-themes, classed as facilitators and barriers. The TDF domains associated with each themes are also presented in Table 4. From the analyses, we found that most of the themes (n = 21/26) were associated with Environmental context and resources domain of the TDF. The subsequent sections of this manuscript describe analyses of each important key themes separately.

**Table 4:** Key themes and subthemes (triangulated with associated TDF domains) as facilitators and barriers

Themes	Subtheme	Sample quote	TDF domains	Facilitator	Barrier
Items predominantly identified as facilitators					
Pharmacist’s perception of their current roles		“The pharmacist must provide the patient with information about their medications, how to take them, what to do if they miss a dose, what to do if the patient has an adverse reaction, answering the patient’s questions and ensure the high-quality use of medicines and advise other healthcare professionals about safe and effective use of medications.” [Female, 20–29 years, hospital pharmacist]	Social professional role and identity; Optimisms; Intentions	✓	
Pharmacist’s perceived definition of non-traditional role in Saudi Arabia	New service that has been established in pharmacy	“I think the new one for me, as I told you, when I saw the anticoagulant clinic, I’ve never seen a clinic that is run by pharmacist.” [Female, 30–39 years, hospital pharmacist]	Knowledge	✓	
How the roles of pharmacists are developing		“The pharmacist role back then was only dispensing medications, but now the pharmacist is more involved with other healthcare professionals in making clinical decisions about patient's care in their scope of practice” [Female, 20–29 years, hospital pharmacist]	Knowledge, Optimism, Environmental context and resources	✓	
Attitude towards progression of pharmacist’s roles	Impact of ease of access to pharmacists on the up-take of non-traditional roles	“I think for the out-patient, some patients need to be highlighted and to be monitored regularly even by phone, or access to the pharmacist to have consultations whenever they need to do so. I think this is the true role of a clinical pharmacists” [Female, 30–39 years, hospital pharmacist]	Skills, Belief about capabilities, Intentions	✓	
Views on the education system to allow the uptake of non-traditional roles	Course structure and focus	“Yes I do believe it is fit. Saudi Arabia pharmacy graduates are trained and exposed to the current pharmacist non-traditional role. After graduating they are fully prepared to lead pharmacist non-traditional role. They are re defining the role of a pharmacist..” [Female, 20–29 years, hospital pharmacist] “Oh yes, pharmacists are taught how to do blood pressure monitoring but also again I’ve never seen this occur to be honest” [Female, 30–39 years, academic]	Knowledge, Environmental context and resources	✓	✓
	Practical experience during degree	“That’s not part of my undergraduate degree and I actually helped developing the training programmes, I actually helped pitch it to our executives, that we need to get more students involved with the Saudi FDA role, even if they don’t work with us, they graduate and they know about us. They can help to educate people.” [Male, 30–39 years, Senior pharmacist, Saudi FDA] “As I say, the PharmD student, they can grow faster than the bachelor, than the pharmB student, because they have different, actually it’s a one year internship but it makes a difference in their life.” [Female, 30–39 years, clinical trials pharmacist]	Skills, Environmental context and resources	✓	✓
Institutional barriers and facilitators	Universities differ in the courses they offer	“Erm, yeh so the curriculum has changed differently from one university to another. But most of them, they focus on the clinical side, how to manage the drug and counsel the patient to use it in the most appropriate way. But, the curriculum changes depending on the update of pharmacy, but also depending on the city and the level of prestige of the university.” [Female, 30–39 years, military hospital pharmacist]	Knowledge, skills and environmental context and resources	✓	✓
	Hospitals differ in recognising clinical pharmacy	“So some hospitals are really developed, they know the concept of clinical pharmacy and they give you the opportunity to work as a clinical pharmacist. However, some hospitals are still stuck to the rules that some pharmacists have to dispense medicines, sometimes just to say a brief description or protocol to the patient, but it depends on the hospital” [Female, 30–39 years, hospital pharmacist]	Social/professional role and identity, Social influences, Environmental context and resources	✓	✓
Sector specific barriers and facilitators		“Yeh, but as I said before the community pharmacy does not provide all of the service. Its not all of the service active like in the hospital.” [Female, 30–39 years, hospital pharmacist] “Because the hospital gives you the opportunity to have direct contact with the patient, to update information in databases, to provide a lot of courses during the month and the year.” [Female, 30–39 years, hospital pharmacist]	Environmental context and resources	✓	✓
Wider support to uptake non-traditional roles	Employer	“Absolutely. We have a tremendous amount of opportunities all year long. From national and international conferences being held in the kingdom to internal staff development programs in hospitals for pharmacy staff. For example, at my hospital we have weekly lectures and updates on pharmacotherapy guidelines.” [Female, 20–29 years, hospital pharmacist]	Environmental context and resources	✓	
	Governmental policies	“So any university, some research related in the hospital, also in research centres which is separate and is supported directly by the government” [Female, 30–39 years, hospital pharmacist]	Environmental context and resources; Behavioral regulations	✓	
Impact of new technology on the uptake of non-traditional roles	Patient records are now computerised and easily accessible	“Now with the technology, its very easy to access, the data can be accessed online or through an archive. I think the technology has really helped the clinical pharmacy to develop their roles.” [Female, 30–39 years, hospital pharmacist]	Environmental context and resources	✓	
Pharmacist’s willingness to develop their own roles	Some pharmacists stick to traditional roles	“I think that there are a lot of sources that pharmacist can look at again it depends on the pharmacist himself or herself err I think there are a lot of sources from the hospital and outside the hospital. Some pharmacist I think, they are very satisfied with traditional concept of pharmacy, dispensing and that’s it and there are a lot of pharmacists that need to develop, and I think the courses are accessible for them.” [Female, 30–39 years, hospital pharmacist]	Intentions		✓
	Some pharmacists take the opportunities to develop	“Yes but I would say, when I graduated from university, I got 40% experience from the university and 60% from the practice itself. So university only gives you the key, so what speciality you want. But the rest you have to decide yourself, you are responsible for this, how to improve yourself.” [Male, 30–39 years, senior hospital pharmacist]	Intentions	✓	
Awareness/knowledge of pharmacist’s non-traditional roles	Patients awareness of the non-traditional roles	“To be honest, I think the patient thinks that pharmacist are only supposed to give them the medication. And tell them how to use it and that’s it. I don’t think they are really educated about the role of pharmacist.” [Female, 30–39 years, hospital pharmacist]	Environmental context and resources; Social influences		✓
	Hospital institutions recognition of non-traditional roles	“hospitals like King Faisal and King Abdulaziz in Jeddah, these are very well developed. They know and acknowledge and know how to apply these concepts of clinical pharmacists.” [Female, 30–39 years, hospital pharmacist] “I think some of the healthcare providers need to be aware of the changes in the pharmacist roles over the years and they should support us. We must make patients' aware of the new pharmacist roles.” [Female, 20–29 years, hospital pharmacist]	Environmental context and resources; Social influences	✓	✓
Items predominantly identified as barriers					
Perceptions on the roles of pharmacists in Saudi Arabia	Patients perceptions on the roles of pharmacists	“most of the pharmacist role is behind the scenes, so reporting adverse drug pharmacovigilance, or there is erm, drug shortage, or this is erm, any clinical trial, the patient usually they are not involved so that can’t see the role of pharmacist in these kinds of scenarios. So that’s why. The only contact point between the patient and the pharmacist when they dispense their medication only.” [Female, 30–39 years, clinical trials pharmacist]	Environmental context and resources; Social influences		✓
	Lack of support from other healthcare professionals	“However, I’ve seen this, when the pharmacist is just dispensing the drug and are calling for a check-up about something with the physician, this is where the clashes happen” [Female, 30–39 years, hospital pharmacist]	Environmental context and resources; Social influences		✓
Pharmacist-patient relationship (trust)	Patients do not trust pharmacists as much as doctors	“so if pharmacists come to a patient to take a blood test or blood glucose level for example, a patient would hesitate. So maybe they can claim that pharmacists have no expertise in this one, so like a fear of the unusual.” [Female, 30–39 years, hospital pharmacist]	Environmental context and resources; Social influences		✓
	Patients who have more contact with pharmacists trust them	“However, some patients who are in chronic disease and require chronic medication and chronic follow up. They acknowledge the role of pharmacists. For example, as I told you, this anticoagulant clinic, when the patient comes, you can really see the trust the patient has for the pharmacist.” [Female, 30–39 years, hospital pharmacist]	Environmental context and resources; Social influences	✓	
Lack of communication between healthcare professionals and pharmacists in community settings		“I think it would be very rare that a pharmacist will just call the GP and ask about it. Its very rare. The role of a pharmacists is just to dispense. If there is a very serious interaction though, that needs to be highlighted, the pharmacy will say it. But other than that, it is very rare that the pharmacist will contact the GP for any enquiries.” [Female, 30–39 years, hospital pharmacist]	Environmental context and resources		✓
Lack of resources	Staff shortage	“there is a shortage of pharmacists and staff. They always complain of this one. So if they are going to do this and apply it in the right way, I think they need to find enough pharmacists for this. To decrease the load for pharmacists, this is the most reasonable solution” [Female, 30–39 years, hospital pharmacist]	Environmental context and resources; Social influences		✓
	Lack of access to patient records	“If we suggest any changes to the plan, and the patient tells the GP later, that would make a conflict. Because I don’t know their history. In this case I would just send them back to their GP. I wouldn’t recommend anything id just send them back.” [Female, 30–39 years, hospital pharmacist with previous experience in community pharmacy]	Environmental context and resources; Social influences		✓
	Lack of consultation rooms	“No, no, I think this is really important, this would be really helpful, really helpful, like even the patient that can feel more secure when they discuss the drug or how to take the medication or to follow-up with the pharmacist. I think the… I saw this here which is really nice, but no unfortunately. This is one of the things that I really wish to have it in the future.” [Female, 30–39 years, academic]	Environmental context and resources; Social influences		✓
Healthcare system related factors	Patients are not registered at community pharmacies	“No no, in community we don’t do that. Because you know patients, they don’t commit with one GP. They tend to go to several hospitals with different GP’s. So the doctor is always changing.” [Female, 30–39 years, hospital pharmacist]	Environmental context and resources; Social influences		✓
	Patients do not obtain their medication from community pharmacies	“But not all of drug, mainly the cosmetic because all of the drugs, they can get in from the hospital. The medical drugs in Saudi Arabia are free from A-Z. The patient no need to take medications from outside but some cosmetic products they need to take from outside so this is the need of community pharmacists, to tell them how to use.” [Female, 30–39 years, military hospital pharmacist]	Environmental context and resources; social influences		✓

### How the roles of pharmacists are developing?

Participants were able to discuss the developing roles and responsibilities of a pharmacists moving away from dispensing and manufacturing roles and made comparisons between current pharmacy practice and pharmacy practice 10–20 years ago.

“Worth to say on the hospital level that they have a better heard voice in the hospital meetings and committees which is great progress as years ago it was only related to the physicians.” [Female, 30–39 years, IV pharmacist].

The change in the pharmaceutical sector was highlighted as a major development and advancement for Saudi Arabia providing pharmacist with the opportunity to work in large pharmaceutical companies and move away from the traditional compounding and manufacturing role to researching and developing drug molecules.

‘…manufacturing role to researching and developing drug molecules: “Erm, actually there are local companies that have been around for more than 20 years but most of the local companies, they did not develop the molecular, they just take it as a powder and prepare it as a different form. But now, the local companies are supported by the government to develop from a small molecule into a drug…” [Female, 30–39 years, military hospital pharmacist].

### Views on the education system to allow the uptake of non-traditional roles

The education system was discussed by participants as an important factor to the development of pharmacists’ roles and providing them with the necessary knowledge and skills to carry out clinical roles. Participants were asked whether the modules taught to undergraduate pharmacy students was fit for purpose to allow pharmacists to uptake non-traditional roles.

“Yes I do believe it is fit. Saudi Arabia pharmacy graduates are trained and exposed to the current pharmacist non-traditional role. After graduating they are fully prepared to lead pharmacist non-traditional role. They are re defining the role of a pharmacist..” [Female, 20- 29 years, hospital pharmacist].

Practical experience and placements throughout the undergraduate years were also identified by participants as a facilitator of the uptake of non-traditional roles as it allowed students to become exposed to real life pharmacy practice and aid in breaking the perception of students that pharmacists’ do not take on roles beyond dispensing.

“As I say, the PharmD student, they can grow faster than the bachelor, than the BPharm student, because they have different, actually it’s a one year internship but it makes a difference in their life.” [Female, 30–39 years, clinical trials pharmacist].

One participant practicing as a senior pharmacist and active member of the pharmaceutical products approval committee felt that students were not gaining practical experience in the Saudi FDA.

“That’s not part of my undergraduate degree and I actually helped developing the training programmes, I actually helped pitch it to our executives that we need to get more students involved with the Saudi FDA role, even if they don’t work with us, they graduate and they know about us. They can help to educate people.” [Male, 30–39 years, Senior pharmacist, Saudi FDA].

### Wider support to uptake non-traditional roles

Participants felt that there was wider support for pharmacists to uptake non-traditional roles, offering continuous professional development opportunities such as training courses and residency programmes.

“Absolutely. We have a tremendous amount of opportunities all year long. From national and international conferences being held in the kingdom to internal staff development programs in hospitals for pharmacy staff. For example, at my hospital we have weekly lectures and updates on pharmacotherapy guidelines.” [Female, 20–29 years, hospital pharmacist].

Wider support from the government was a sub-theme identified from the transcripts. Participants felt that the government encouraged them to uptake non-traditional roles particularly through initiatives of Saudi Vision 2030.

“Recently, there a lot of things changing. The first one is to push all young people to work as a manager, as a leader, which is good. Prince Mohammed Bin Salman, he is pushing all of the young people, really supporting them from A-Z.” [Female, 30–39 years, military hospital pharmacist].

However, one participant, a clinical trials pharmacist described that there was generally a lack of recognition of some of the roles such as involvement in research or regulatory affairs.

“but until now they don’t erm, they don’t classify, or they don’t consider any other non-traditional career paths like research, like pharmacists involved in research or pharmacoeconomics, or regulatory affair or drug pricing, pharmacovigilance.” [Female, 30–39 years, clinical trials pharmacist].

### Awareness/knowledge of pharmacist’s non-traditional roles

The importance of the awareness and knowledge of non-traditional roles within pharmacy was identified as key to the application of these roles within different institutions. For example, one participant highlighted that being aware of clinical pharmacy practice was a facilitator to the uptake of non-traditional roles.

“Hospitals like King Faisal [Specialist] and King Abdulaziz [Medical City] in Jeddah, these are very well developed. They know and acknowledge and know how to apply these concepts of clinical pharmacists.” [Female, 30–39 years, hospital pharmacist].

However, one participant described a lack of knowledge on clinical roles, particularly by patients.

“To be honest, I think the patient thinks that pharmacists are only supposed to give them the medication. And tell them how to use it and that’s it. I don’t think they are really educated about the role of pharmacist.” [Female, 30–39 years, hospital pharmacist].

### Perceptions on the roles of pharmacists in Saudi Arabia

Patients’ perceptions on the roles and what services they expect from pharmacists in Saudi Arabia was identified by all participants. Participants felt that patients were not as comfortable with pharmacists when enquiring about their healthcare as they were with doctors which may be due to lack of awareness on the development of clinical pharmacy practice.

“Until now, a patient expect pharmacist to know every single thing about drugs, even about herbal medication. They even come and ask us whether I can use this herbs instead of this drug or not. So everything people expect from us is knowing about drugs. When it comes to disease or diagnosis, they tend not to listen to us. They prefer to go to the physician or GP.” [Female, 30–39 years, hospital pharmacist].

The lack of support from other healthcare professionals due to perceptions and expectations of the roles of pharmacists was another sub-theme identified regarding key challenges towards the uptake of non-traditional roles by pharmacists. Participants paid attention to the fact that many healthcare professionals do not recognise the clinical knowledge of pharmacists and are reluctant to take on board a suggestion from a pharmacist.

“However, I’ve seen this, when the pharmacist is just dispensing the drug and are calling for a check-up about something with the physician, this is where the clashes happen” [Female, 30–39 years, hospital pharmacist].

However, as highlighted by another participant, this perception was different where a pharmacist was regularly involved in ward rounds and works as part of the multidisciplinary team when reviewing a patient.

“I think this is most of the time when the pharmacist really works on a daily basis and er, are in the wards going with the physician on the daily rotation, I think they will develop a good relationship with them and they work easily.” [Female, 30–39 years, hospital pharmacist].

### Impact of new technology on the uptake of non-traditional roles

The increase in the use of technology within healthcare was described by participants as a facilitator to uptake non-traditional roles particularly due to easy access to patient records and prescriptions. This would allow pharmacists to intervene when necessary and take a holistic approach to the treatment of patients.

“Now with the technology, its very easy to access, the data can be accessed online or through an archive. I think the technology has really helped the clinical pharmacy to develop their roles.” [Female, 30–39 years, hospital pharmacist].

### Pharmacist–patient relationship (trust)

Participants identified the relationship between the pharmacist and the patient as having a key impact on a pharmacist’s ability to carry out non-traditional roles. Participants felt that currently, many patients do not trust the pharmacists due to pharmacists not being able to develop their relationship with them further.

“So patients, they don’t accept the interventions done by pharmacists. They say ok, this medication has been prescribed for me from the doctor, and the doctor knows what they are doing, and they feel that the doctor knows everything. So they don’t accept pharmacists to take a bigger role.” [Male, 30–39 years, senior hospital pharmacist].

One participant explained that where a patient is in a chronic condition and is required to meet with the pharmacist often, they begin to develop a strong relationship with them and are recognised by the patient as a key healthcare provider.

“However, some patients who are in chronic disease and require chronic medication and chronic follow up. They acknowledge the role of pharmacists. For example, as I told you, this anticoagulant clinic, when the patient comes, you can really see the trust the patient has for the pharmacist.” [Female, 30–39 years, hospital pharmacist].

## Discussion

To authors’ knowledge, this is the first qualitative study that has explored the pharmacists’ perception about the uptake of non-traditional roles in pharmacy in Saudi Arabia. The findings of this study suggest that there is a clear shift towards the uptake of non-traditional pharmacy roles with participants showing an overall positive attitude towards the way pharmacy practice is progressing in Saudi Arabia.

Participants defined their current roles as pharmacists as being consultants of medicines and were expected to recall all information on a drug. This correlates with the current perceptions of patients about the roles of pharmacists and what services a patient expects pharmacists to provide. Participants were unanimous on their views about patient expectations and believed that patients were not willing to seek advice from community pharmacists on their disease states or general issues regarding their healthcare. Previous research has shown that community pharmacists in Saudi Arabia are often expatriate males [32–34] and therefore there may often be language or cultural barriers to patients advice seeking from pharmacy [35]. The new system of the Ministry of Health Wasfaty and the nationalisation employment program in community pharmacies is aimed to address these barriers [36, 37].

Participants attributed the lack of patient engagement to the limited clinical knowledge of community pharmacists. However, participants who stated hospital pharmacy as their current clinical practice setting, reported that their regular patients had a different view on the role of pharmacists and felt that patients perceived them to be an integral member of the multidisciplinary healthcare team. Previous research shows that majority of Saudi pharmacy students showed interest to work in hospitals after graduation [38]. Taking a holistic, patient-centred approach is important to allow patients to recognise pharmacists as healthcare providers [39].

Participants were able to provide examples of non-traditional roles of pharmacists which existed in their respective practice setting. Pharmacists running their own specialised clinics such as anticoagulant and diabetes clinic was amongst the examples of non-traditional roles cited by participants. Other non-traditional roles quoted by participants included pharmacists in health informatics; clinical trials and pharmacists working in specialised ambulatory clinics. Wider support available for pharmacists to uptake non-traditional roles was perceived positively by participants. Hospitals such as King Faisal Specialist Hospital & Research centre are now offering the PGY-1 and PGY-2 residency programmes to train pharmacists to specialise and become clinical pharmacists in their chosen area of interest [40]. Majority of the participants felt that there was support from the Ministry of Health for pharmacists to carry out continuous professional development. In order to keep professional license valid, every pharmacist has to get not less than 20 CPD credits a year in Saudi Arabia.

Lack of exposure to patients and real-life pharmacy practice experience during pharmacy education was identified as a barrier towards the uptake of non-traditional roles by participants. The introduction of the PharmD’s one-year internship, however, was described by participants as a positive change in the education system as it allowed students to experience various clinical pharmacy sectors during their rotations in hospitals.

Participants were asked to identify any major challenges towards the uptake of non-traditional roles by pharmacists in Saudi Arabia. Lack of support and reluctance to acknowledge the pharmacist interventions and suggestions for improving patient healthcare were among some of the challenges highlighted by participants. A previous study that explored the perceptions of physicians about the clinical roles of pharmacist in hospitals in Saudi Arabia reported that only 4% of the physicians had worked with a clinical pharmacist previously while majority were unclear about the role of a clinical pharmacist [41]. Lack of communication between physicians and pharmacists particularly in community settings was reported by majority of the participants who expressed that pharmacists did not communicate with the doctors in routine unless necessary such as communicating about a serious interaction on the prescription. Communication gaps between healthcare institutions and professionals has been recognised as a contributor towards medication safety problems seen in Saudi Arabia [42]. Lack of consultation rooms, the lack of access to patient care records and staff shortages were some of the other challenges that were highlighted by the participants. These findings corroborates with previous studies investigating practice changes in community pharmacy [43–46] and in Saudi Arabia these factors could be related to deficiency of experiential part of training, lack of clinically qualified preceptors, lack of training in advanced settings as were addressed in published studies [47–49]. Participants believed that such barriers limited pharmacists’ roles and if addressed, would support the uptake of non-traditional roles as well as improve patient health outcomes. There is a need to raise patient awareness about the non-traditional roles of pharmacists in all practice settings. It may be achieved through provision of patient education about the clinical roles of pharmacists. The successful implementation of the initiatives set out in the new Model of Care as part of Saudi Vision under the Ministry of Health will enhance the efficacy of the current healthcare system and will have a significant impact on the development of community pharmacy practice in Saudi Arabia. Furthermore, increasing the workforce of Saudi pharmacists in community pharmacies will help resolve the issue of staff shortages in community settings.

The expansion of pharmacists’ roles and workforce in the community is also imperative on the face of the COVID-19 pandemic and other infectious diseases of concern [50–53]. The pandemic has required pharmacy services to offer extended roles in all sectors including continuation of the provision of routine services in the time of hardships and shortages in medicines supply; uptake of novel roles in primary care, hospital and critical care; participation in clinical trials; and provision of education, medicines information; and offering vaccinations [50]. The findings of this study will be useful in enabling pharmacists’ preparedness and response to current and future pandemic in Saudi Arabia and beyond by harnessing the facilitators and addressing barriers to uptake of novel roles as identified in this study.

The use of TDF to the research is a key strength to this study. The domains identified from the TDF analysis can be used to map the appropriate behaviour change techniques (BCTs) using behavioural change wheel (BCW to design the appropriate interventions to address the barriers identified in this study [21, 54]. Recruitment through convenience sampling, non-representation of all sectors of practice, and lack of data saturation are key limitations. However, the participants represented experienced practitioners with insight of pharmacy practice in both Saudi Arabia and a Western country with advanced pharmacy practice roles. A larger qualitative study and interventions development incorporating novel roles and their evaluations are needed that can inform policy makers, pharmacy services, researchers, and health systems in Saudi Arabia to further the uptake of non-traditional roles by pharmacists.

## Conclusions

This theoretically informed qualitative study shows that there is a growing interest in pharmacists towards the uptake of non-traditional roles in Saudi Arabia. Pharmacists showed an overall positive attitude towards the way pharmacy practice is progressing in Saudi Arabia. However, there is a need for some interventions especially those aiming to modify environmental context and resources to encourage Saudi pharmacists to update the non-traditional roles. Addressing such barriers and promoting uptake of novel roles by pharmacists is imperative in the context of COVID-19 and future pandemics.

## Data Availability

All data generated or analysed during this study are included in this published article.

## Abbreviations

MOH: Ministry of Health
OTC: Over-the-counter
TDF: Theoretical Domains Framework
UK: United Kingdom
